# A study protocol for a feasibility randomised controlled trial investigating videoendoscopic radical inguinal lymphadenectomy versus open radical inguinal lymphadenectomy in patients with penile cancer (VELRAD)

**DOI:** 10.1186/s40814-024-01474-8

**Published:** 2024-04-10

**Authors:** Stanley Tang, Clare Akers, Hussain Alnajjar, Ben Ayres, Cinzia Baldini, Andrew Embleton-Thirsk, Kurinchi Gurusamy, Paul Hadway, Vivekanandan Kumar, Maurice Lau, Raj Nigam, Karl Pang, Arie Parnham, Elena Pizzo, Veronica Ranieri, Rowland Rees, Vijay Sangar, Anvi Wadke, Norman Williams, Asif Muneer

**Affiliations:** 1 University College London Hospitals NHS Trust, London, UK; 2 St George’s University Hospitals NHS Trust, London, UK; 3 University College London, London, UK; 4 Royal Berkshire NHS Foundation Trust, Reading, UK; 5 Norfolk and Norwich University Hospitals NHS Foundation Trust, Norwich, UK; 6 The Christie NHS Foundation Trust, Manchester, UK; 7 Royal Surrey County Hospital, Surrey, UK; 8 University Hospital Southampton NHS Foundation Trust, Southampton, UK

**Keywords:** Penile cancer, Lymphadenectomy, Squamous cell carcinoma, Melanoma

## Abstract

**Background:**

Penile cancer is a rare male genital malignancy. Surgical excision of the primary tumour is followed by radical inguinal lymphadenectomy if there is metastatic disease detected by biopsy, fine needle aspiration cytology (FNAC) or following sentinel lymph node biopsy in patients with impalpable disease. However, radical inguinal lymphadenectomy is associated with a high morbidity rate, and there is increasing usage of a videoendoscopic approach as an alternative.

**Methods:**

A pragmatic, UK-wide multicentre feasibility randomised controlled trial (RCT), comparing videoendoscopic radical inguinal lymphadenectomy versus open radical inguinal lymphadenectomy. Patients will be identified and recruited from supraregional multi-disciplinary team meetings (sMDT) and must be aged 18 or over requiring inguinal lymphadenectomy, with no contraindications to surgical intervention for their cancer. Participants will be followed up for 6 months following randomisation. The primary outcome is the ability to recruit patients for randomisation across all selected sites and the rate of loss to follow-up. Other outcomes include acceptability of the trial and intervention to patients and healthcare professionals assessed by qualitative research and obtaining resource utilisation information for health economic analysis.

**Discussion:**

There are currently no other published RCTs comparing videoendoscopic versus open radical inguinal lymphadenectomy. Ongoing study is required to determine whether randomising patients to either procedure is feasible and acceptable to patients. The results of this study may determine the design of a subsequent trial.

**Trial registration:**

Clinicaltrials.gov PRS registry, registration number NCT05592639. Date of registration: 13th October 2022, retrospectively registered

**Supplementary Information:**

The online version contains supplementary material available at 10.1186/s40814-024-01474-8.

## Background

Penile cancer (PeCa) is a rare male genital malignancy. A total of 637 cases of penile cancer were recorded in the UK in 2015 with approximately 134 deaths in 2016 [1]. However, the incidence of PeCa in Western countries is increasing [2–4]. Squamous cell carcinoma is the most common histological subtype and accounts for more than 90% of penile cancers [3, 4]. Other cancers of the penis include mucosal melanoma and sarcoma [3].

The surgical management depends on the stage of the disease [5]. Localised tumour can undergo penile preserving surgery (PPS) which includes glans resurfacing (PeIN, T1), glansectomy (T2), and partial penectomy (T3). Total penectomy is a non-organ preserving procedure, reserved for locally advanced PeCa [6].

The presence of metastatic disease in the inguinal lymph nodes is one of the most important prognostic indicators in PeCa [7, 8]. Up to 25% of PeCa patients with impalpable inguinal lymph nodes may harbour micrometastatic disease [8]. The sensitivity of CT, MRI, and ultrasound-guided fine needle aspiration (FNAC) is suboptimal to detect micrometastases [6, 8]. Therefore, dynamic sentinel lymph node biopsy (DSLNB) or superficial modified inguinal lymphadenectomy with frozen section analysis are the main treatment options for lymph node management in patients with impalpable inguinal lymph nodes. High risk tumours include tumour stage >T1b (tumour exhibits lymphovascular invasion and/or perineural invasion or is high grade, i.e. grade 2/3 or sarcomatoid subtype) [6, 8]. If there is metastatic disease in the sentinel lymph node, radical inguinal lymphadenectomy is recommended, as this has been shown to improve cancer-specific survival [6, 8]. Open inguinal lymphadenectomy is an option if DSLNB is unavailable or unable to be performed; for example in patients who have undergone a total penectomy, have non-visualisation on dynamic sentinel node imaging or if nuclear medicine facilities are unavailable. Surveillance may be applicable in selected patients with T1a disease or less with clinically impalpable inguinal nodes. In men with PeCa and palpable inguinal nodes, FNAC or open biopsy followed by radical inguinal lymphadenectomy is the recommended treatment [6, 8].

### Aims

The aim of the study is to assess the feasibility of performing a RCT comparing videoendoscopic inguinal lymphadenectomy versus open radical inguinal lymphadenectomy in men with penile or urethral cancer requiring inguinal lymphadenectomy, and determine the design of a definitive RCT.

## Rationale

### Justification for a randomised controlled trial

It is important to evaluate the effectiveness and cost-effectiveness of VEIL versus open radical inguinal lymphadenectomy in men with PeCa. The effectiveness from observational studies may be influenced by selection bias, i.e. people with smaller and mobile nodes would have been chosen for VEIL, while those with large and fixed nodes would have been chosen for open lymphadenectomy. RCTs overcome this risk of selection bias. The lack of major completed or ongoing trials shows that well-designed studies are necessary.

### Justification for a feasibility study

In the only previously attempted RCT which compared VEIL versus open inguinal lymphadenectomy for multiple cancers (but mainly melanomas), randomisation could not be performed because of patient preference [9]. This was despite the absence of information on long-term oncologic outcomes and increased incidence of lymphocele in patients undergoing VEIL versus open inguinal lymphadenectomy for PeCa. The primary outcome in that RCT was wound complications [9]. However, our survey in the PeCa patient support group indicated that people rated complications differently, suggesting that some complications are more important than others. Therefore, it is important to judge the acceptability of the trial to patients and clinicians, the acceptance of randomisation, estimate the recruitment rate, informing the primary outcome of a definitive trial, and the difference in the primary outcome that can be considered clinically meaningful. A feasibility study is therefore necessary to assist in the design of a definitive RCT.

## Methods

### Study design

This is a pragmatic, UK-wide multicentre feasibility RCT, involving four NHS Foundation Trusts (University College London Hospitals, The Christie Hospital, Norfolk and Norwich University Hospitals, St George’s University Hospitals).

Patients requiring inguinal lymphadenectomy who fulfil the inclusion/exclusion criteria (see Table 1) will be identified during the sMDT meetings and in outpatient clinics.

**Table 1:** Inclusion and exclusion criteria

Inclusion criteria	1. Patients requiring inguinal lymphadenectomy
	a) Patients with squamous cell carcinoma or mucosal melanoma of the penis > T1bG2 or patients with urethral cancer requiring inguinal lymphadenectomy
	b) Patients unsuitable for dynamic sentinel node biopsy (DSNB) with impalpable nodes (previous penectomy or non-visualisation at previous DSNB)
	c) Previous DSNB with metastatic inguinal nodes on histology or FNA-positive nodes on cytology who require a completion radical inguinal lymphadenectomy
	d) Small volume palpable inguinal lymph nodes (< 2 cm on CT) not fixed to skin
	2. Aged > 18 years
Exclusion criteria	1. Unfit for surgery
	2. People unlikely to benefit from lymphadenectomy because of advanced cancer
	3. Those with palpable inguinal lymph nodes fixed to skin or adjacent structures
	4. Does not want to participate in the trial or unable to provide informed consent

### Trial outcomes

The aim is to assess the feasibility of performing a RCT comparing videoendoscopic inguinal lymphadenectomy versus open radical inguinal lymphadenectomy in men with penile or urethral cancer requiring inguinal lymphadenectomy, and determine the design of a definitive RCT.

The primary outcome for this trial will be the feasibility of the RCT as assessed by:

- Ability to recruit patients across all participating sites (recruitment rate). We aim to recruit (randomise) > 30% (0.3) of eligible patients approached.
- Acceptability of the trial design and intervention to both patients and healthcare professionals through semi-structured interviews.

Recruitment rate will be monitored through screening and randomisation logs with the aim to recruit approximately three patients per month at all four sites.

There is currently no core outcome set for trials on patients with PeCa. Patient representatives and clinicians have identified complete removal of the cancer, different types of complications, successful completion of VEIL without requiring conversion to open lymphadenectomy, overall survival, health-related quality of life, length of hospital stay, return to normal activity, and the number of workdays lost (in those who work) as important outcome measures. These will be considered in this feasibility trial to help in the design of a definitive trial. We will measure these outcomes at 6 months after surgery.

### Randomisation and blinding

Randomisation will be performed using a web-based platform via pre-prepared lists from the Trial Statistician with stratification by the centre and stage of cancer (I or II) and a block size of 2 or 4. The randomisation schedule will be concealed from all members of the clinical teams.

It is not possible to blind the healthcare providers or patients to the treatment received. Blinding of patients is not possible post-operatively. We do not anticipate any outcome measured within this period as the primary outcome of the trial. We will attempt to blind the outcome assessors of the long-term complications and oncologic outcomes. Lack of blinding will not result in a bias of the primary outcomes of the trial, namely assessment of feasibility of a definitive RCT as assessed by ability to recruit patients at the selected sites (recruitment rate) and acceptability of the trial/intervention to patients and healthcare professionals.

### Intervention

The trial compares videoendoscopic radical inguinal lymphadenectomy (intervention) versus open radical inguinal lymphadenectomy (control).

Surgical technique is based on individual surgeons; however all centres involved in the trial carry out high-volume penile cancer surgery, including inguinal lymph node dissection. Multiple techniques exist for open inguinal lymphadenectomy, although all techniques remove the inguinal lymph nodes in both the superficial and inguinal group.

### Consenting

In a previous RCT comparing VEIL and open inguinal lymphadenectomy for melanoma in the USA, there was patient preference for VEIL. Given VEIL in the treatment of PeCa is established but not yet standard in the UK, we do not anticipate patient preference for VEIL. Should the participant indicate preference for either intervention at approach, we will offer randomisation and reiterate that the outcome represents the intention to treat with that intervention.

Information about the trial is given to patients face-to-face and in the form of a patient information leaflet. Only clinical staff trained in research delivery as evidenced by completion of Good Clinical Practice training will approach potential participants. Patient information leaflets have been reviewed and approved by the chief investigator, the trial team and the oversight committee. Patients are then given a minimum of 24 h to comprehend the information, with opportunity given for clarification, before deciding whether to participate in the RCT.

### Follow-up and timing of measurement of outcomes

**Fig. 1:**
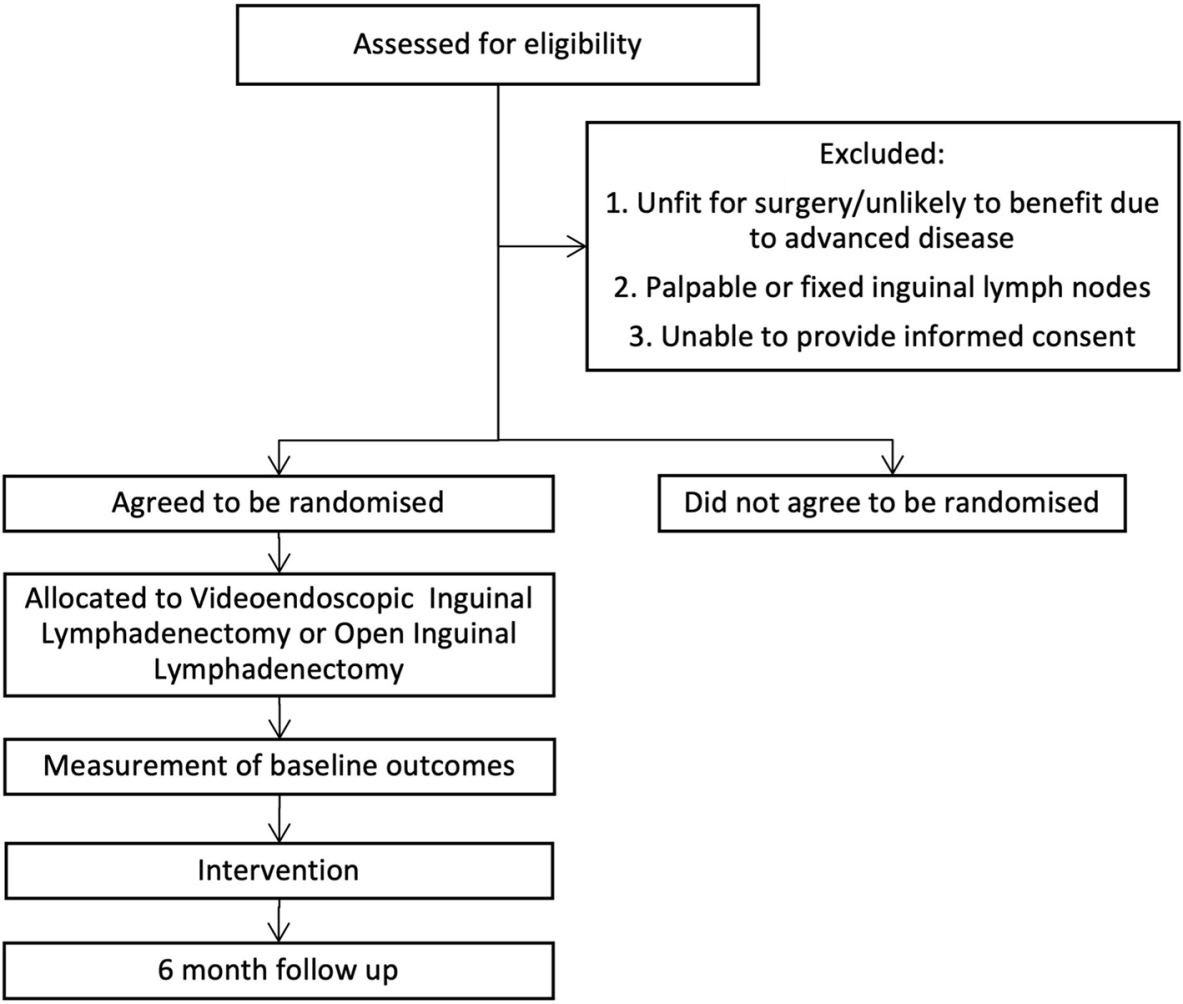
Participant flow chart

Participants will be followed up for 6 months from randomisation. Outcomes will be measured at randomisation, 1 week, 3 to 4 weeks, 3 months, and 6 months (see Fig. 1 and Table 2).

**Table 2:** Assessment and visit schedule (SPIRIT figure)

Time point	Pre-intervention		Intervention phase	Follow up phase			
Assessments	Screening	Baseline		1 week	3–4 weeks	3 months	6 months
Inclusion and exclusion criteria	X						
Consent	X						
Registration		X					
Randomisation	X						
Demographic data		X					
Medical history		X					
Concomitant medications		X					
Tumour characteristics (including stage)		X					
CT scan						X	X
Outcome data				X	X	X	X
AE reporting			X	X	X	X	X

### Qualitative research

We will conduct semi-structured interviews informed by a topic guide developed from the existing literature in conjunction with the research management committee which includes patient representatives [10].

With regard to recruitment, we will explore the reasons for participation and non-participation of patients and patients’ and clinicians’ acceptability of the trial. Non-participation can be related to how information is presented to the patient and the patient’s understanding of the information [11–13].

Participants will be divided into two groups according to whether they agreed or did not agree to be randomised to the trial. The interviews will explore patients’ perspectives of the treatment, their understanding of the two treatments, their reasons for taking part or refusing the trial, and their acceptability of randomisation between the procedures. We will recruit patients who refused randomisation, those who consented, and those who withdrew consent. Recruitment to the interviews will take place alongside recruitment to the trial.

### Sample size justification

The aim of this study is to assess the feasibility of performing a subsequent large definitive RCT on comparing videoendoscopic inguinal lymphadenectomy versus open radical inguinal lymphadenectomy in men with penile or urethral cancer. Therefore, sample size calculations are less relevant.

We aim to approach 170 eligible patients and ask for their consent to be randomised into the trial. We anticipate that the approximate recruitment (randomisation) rate will be 30%. As such, 170 patients would be sufficient to be able to estimate the consent rate to within ±7% through construction of 95% confidence interval for the recruitment rate. Under the assumption that the recruitment rate is approximately 30%, we anticipate that up to 50 patients will be randomised (1:1 VEIL: standard approach) to take part in the study. Sample sizes between 24 and 50 have been recommended to estimate the standard deviation required for a sample size calculation to allow us to design a large RCT aimed at evaluating the cost-effectiveness of videoendoscopic inguinal lymphadenectomy versus open radical inguinal lymphadenectomy in men with penile or urethral cancer requiring inguinal lymphadenectomy [14, 15].

### Patient and public involvement

Our oversight committee comprises of an external Consultant Urologist and a designated patient representative. The committee will be consulted throughout the trial and have access to relevant trial documents.

## Statistical analysis

### Quantitative analysis

Since this is a feasibility study, all analyses other than recruitment rate should be considered exploratory. Acceptability of the trial design to both patients and healthcare professionals will be assessed. Any exploratory comparisons will use means and standard deviations or medians and inter-quartile ranges for continuous variables, as appropriate, and frequency counts and percentages for categorical variables. The proportion of patients who consent to be randomised will be presented with a 95% confidence interval. For other outcomes, we will explore the mean difference in proportion (for binary outcomes) and mean or median difference (continuous outcomes) between the two groups and will be presented with associated 95% confidence intervals. No pre-specified formal comparisons between the groups will be made and no hypothesis tests will be carried out.

Other outcomes which may be considered are complete removal of the cancer, different types of complications, successful completion of VEIL without requiring conversion to open lymphadenectomy, overall survival, health-related quality of life, length of hospital stay, return to normal activity, and the number of workdays lost (in those who work). The randomisation stratification factors of centre and stage of cancer will be considered adjusting factors where appropriate. The results will inform us how sensitive the outcome measures are and, along with other information, will be used to determine the primary outcome of a subsequent large RCT. These results will inform the calculations for a sample size calculation for the most appropriate primary outcome. Any missing data mechanisms will be summarised from the VELRAD trial in preparation for a follow-on trial.

### Qualitative analysis

Interviews will be transcribed verbatim and managed and coded using NVivo Computer Assisted Qualitative Data Analysis Software (i.e. NVivo). Braun and Clarke’s model of thematic analysis with a six-phase approach will be used to generate, review, and define themes within the interview transcripts [16]. The transcripts will be analysed by two researchers. The first researcher will fully code and analyse all transcripts. A sample of these will then be analysed by a second researcher. Themes will be double-checked by the second researcher, and any disagreements will be discussed and resolved within the qualitative research team. Information gained from the process evaluation will be used to inform the development of the protocol for a definitive trial.

### Health economic analysis

We will undertake a feasibility study for an economic evaluation to compare VEIL with open inguinal lymphadenectomy in men with penile or urethral cancer. The feasibility study will be used to plan the evaluation within a subsequent trial, which will aim to estimate the incremental cost of the VEIL and the potential benefit in economic terms from the NHS perspective and personal social services (PSS) perspective versus open inguinal radical lymphadenectomy in men with penile or urethral cancer. We will also explore the feasibility of collecting data to assess the costs for patients and families from a broader perspective.

With the available data, we will perform a preliminary analysis of the cost-utility of VEIL with open inguinal lymphadenectomy in men with penile or urethral cancer to inform a future trial.

## Discussion

Open radical inguinal lymphadenectomy is a procedure with a high short-term and long-term morbidity. Consultation with a Penile Cancer Patient Support group at the host institution indicated that for 9/9 (100%) of patients completing the questionnaire, complications of the procedure were one of the top three most important factors they considered in relation to undergoing lymphadenectomy. The proposed comparison of methods of lymphadenectomy (videoendoscopic inguinal lymphadenectomy versus open inguinal lymphadenectomy) was discussed in the PeCa patient support group in April 2018, and most patients felt this was an important topic to be researched.

Between 20 and 60% of patients undergoing radical lymphadenectomy have one or more of the following complications: lymphoedema (lower limb or genital), lymphocele, wound infection, wound dehiscence, flap necrosis, recurrent cellulitis or deep vein thrombosis [6, 8, 17]. Videoendoscopic inguinal lymphadenectomy may decrease these complications compared to open radical lymphadenectomy and provide equivalent short-term oncological outcomes. However, this is based on non-randomised studies and the long-term oncological outcomes are unknown [18]. There is also concern regarding the higher risk of lymphocele, which is usually asymptomatic, but can be painful and require treatment in about 4 to 7% of people [6, 19]. In the only attempted RCT comparing VEIL versus open radical inguinal lymphadenectomy, it was difficult to recruit because of patient preference for VEIL in the USA. The majority of patients in this trial were also patients diagnosed with melanoma [9]. In addition, non-participation can be related to how information pertaining to the clinical trial is presented to the patient, and the patient’s understanding of the information [11–13]. There are currently no other published RCTs comparing VEIL versus open radical inguinal lymphadenectomy.

We searched the ClinicalTrials.gov and WHO ICTRP (World Health Organization International Clinical Trials Registry Platform). There is only one ongoing RCT involving patients with PeCa undergoing lymphadenectomy [20]. This trial (InPACT) is being conducted in the UK as well as North America and other South American centres to investigate the role of neoadjuvant treatment in large inguinal nodes along with inguinal lymphadenectomy and the role of pelvic node dissection in patients with PeCa [20]. This trial is recruiting patients with larger volume nodal disease requiring neoadjuvant chemo-radiation and will therefore not compete with the current trial which is for non-palpable or small volume nodes. There is currently no ongoing RCT comparing VEIL versus open radical inguinal lymphadenectomy in patients with PeCa worldwide.

### Supplementary Information

**Supplementary material 1.**

## Data Availability

Data sharing is not applicable to this article as no datasets were generated or analysed during the current study.
